# Supplementary figures and images for: The European and Japanese outbreaks of H5N8 derive from a single source population providing evidence for the dispersal along the long distance bird migratory flyways
Source: PeerJ. 2015 Apr 30;3:e934. doi: 10.7717/peerj.934 (PMC4419530; doi:10.7717/peerj.934)

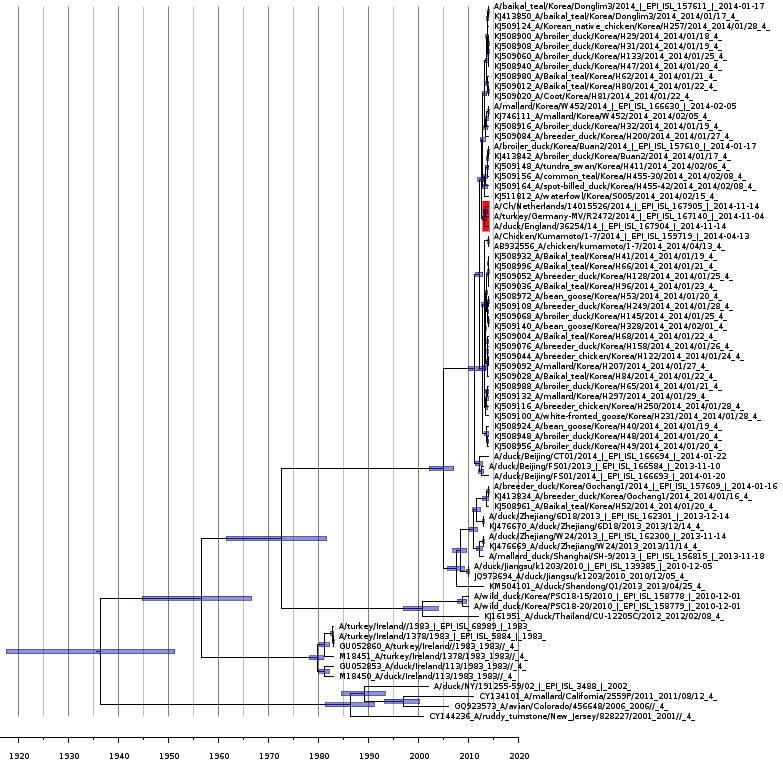

Supplement: Figure S1 — Bayesian coalescent gene tree for all of the H5N8 hemagglutinin segment sequences. The blue bars on the nodes represent the 95% highest posterior densities of the branch heights (this is the time for divergence in months). The European and Japanese clade is highlighted in red. The x-axis represents the date in years. [file peerj-03-934-s001.png]

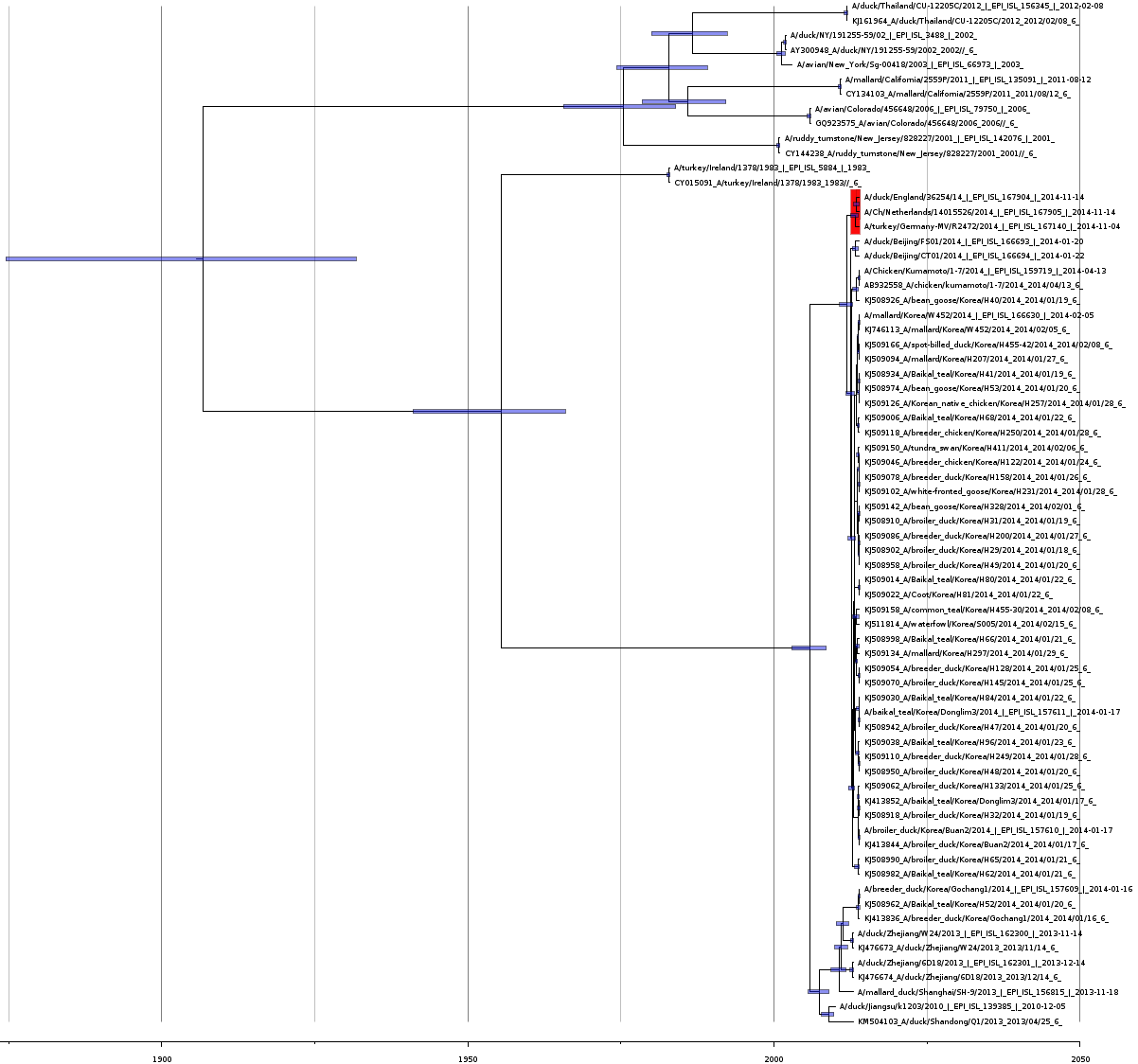

Supplement: Figure S2 — Bayesian coalescent gene tree for all of the H5N8 neuraminidase segment sequences. The blue bars on the nodes represent the 95% highest posterior densities of the branch heights (this is the time for divergence in months). The European and Japanese clade is highlighted in red. The x-axis represents the date in years. [file peerj-03-934-s002.png]

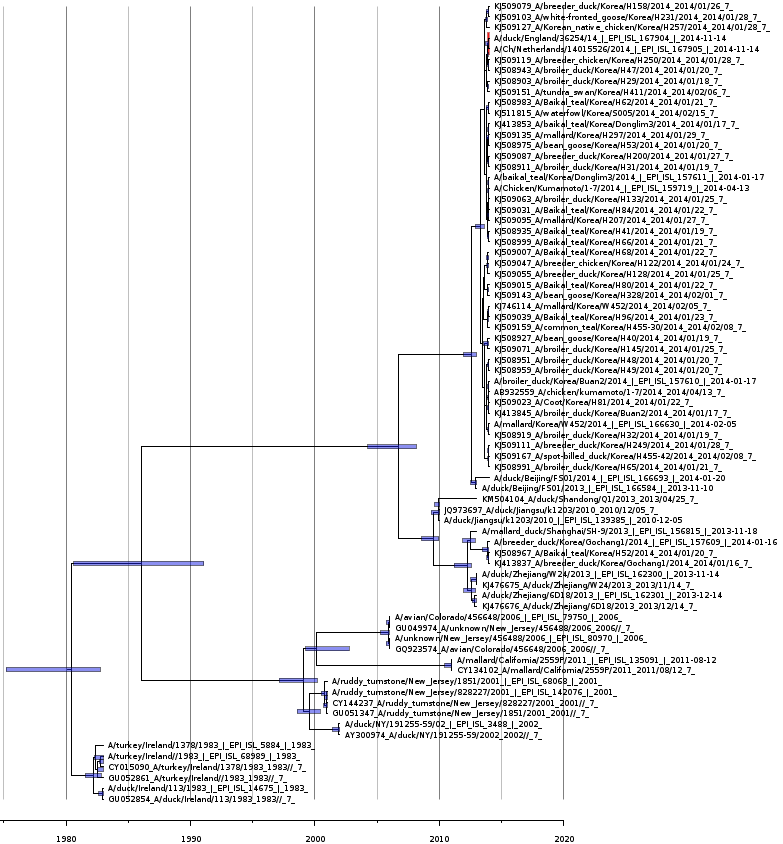

Supplement: Figure S3 — Bayesian coalescent gene tree for all of the H5N8 matrix protein segment sequences. The blue bars on the nodes represent the 95% highest posterior densities of the branch heights (this is the time for divergence in months). The European and Japanese clade is highlighted in red. The x-axis represents the date in years. [file peerj-03-934-s003.png]

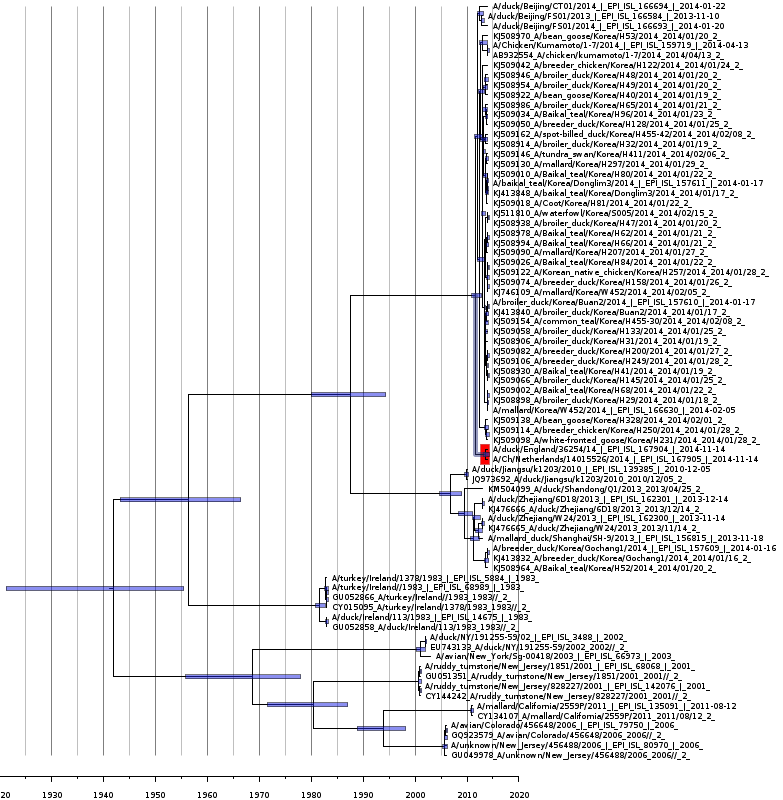

Supplement: Figure S4 — Bayesian coalescent gene tree for all of the H5N8 polymerase subunit (PB1) segment sequences. The blue bars on the nodes represent the 95% highest posterior densities of the branch heights (this is the time for divergence in months). The European and Japanese clade is highlighted in red. The x-axis represents the date in years. [file peerj-03-934-s004.png]

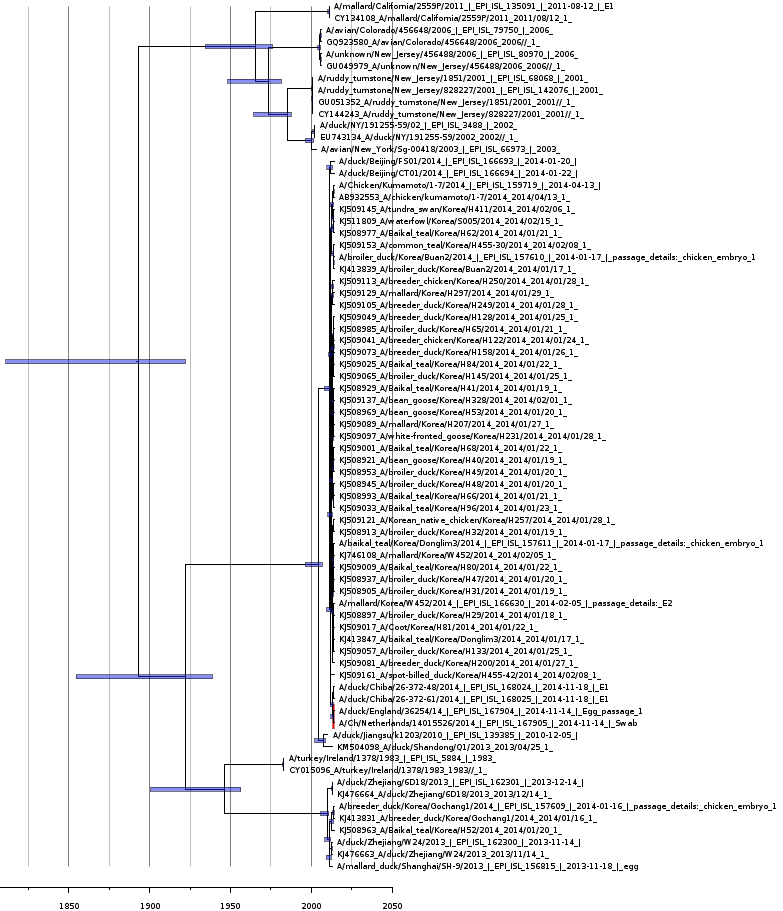

Supplement: Figure S5 — Bayesian coalescent gene tree for all of the H5N8 polymerase subunit (PB2) segment sequences. The blue bars on the nodes represent the 95% highest posterior densities of the branch heights (this is the time for divergence in months). The European and Japanese clade is highlighted in red. The x-axis represents the date in years. [file peerj-03-934-s005.png]

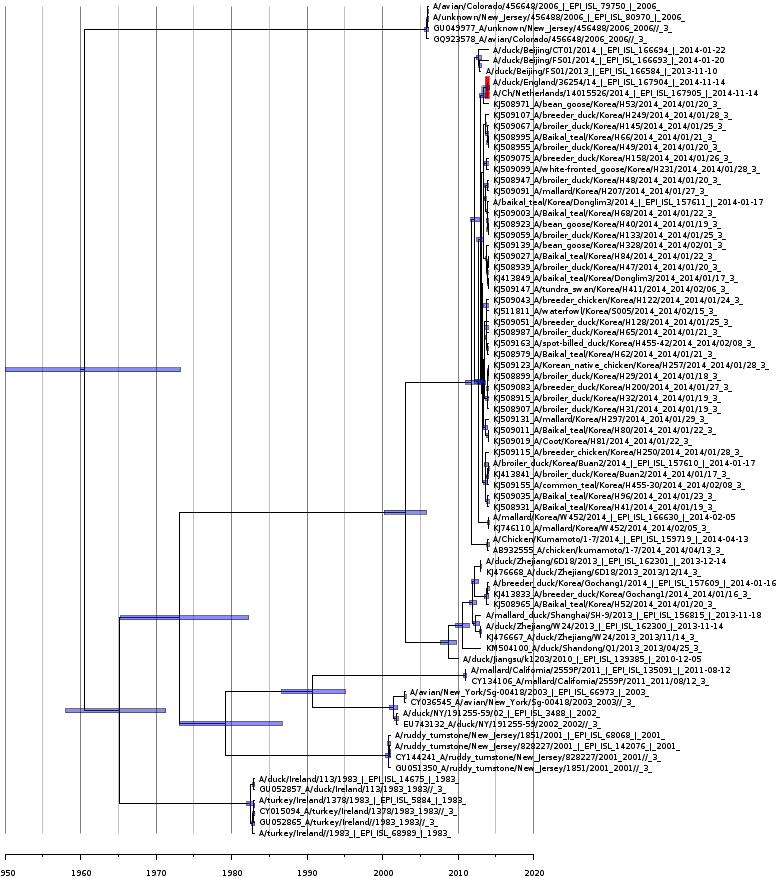

Supplement: Figure S6 — Bayesian coalescent gene tree for all of the H5N8 polymerase subunit (PA) segment sequences. The blue bars on the nodes represent the 95% highest posterior densities of the branch heights (this is the time for divergence in months). The European and Japanese clade is highlighted in red. The x-axis represents the date in years. [file peerj-03-934-s006.png]

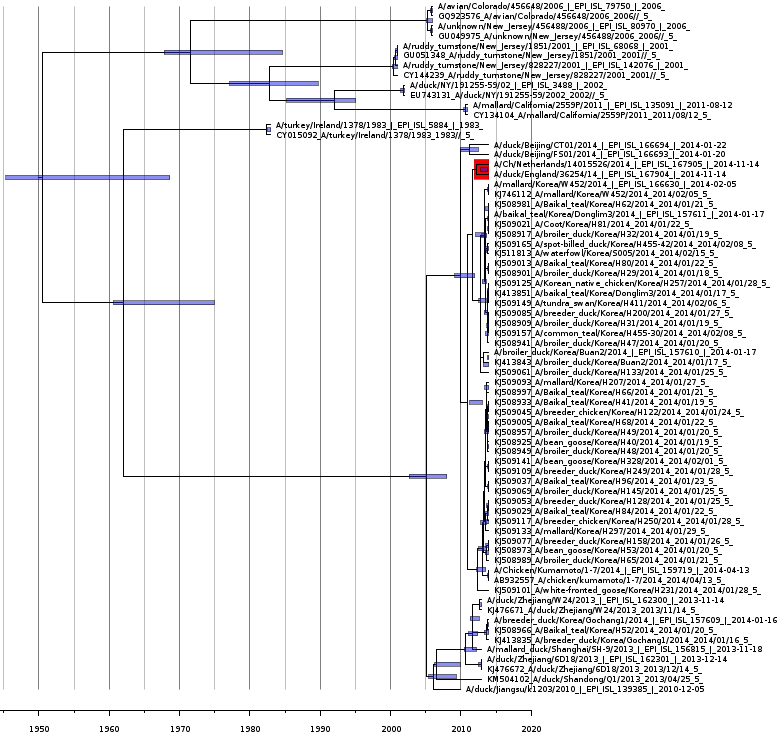

Supplement: Figure S7 — Bayesian coalescent gene tree for all of the H5N8 nucleoprotein subunit (NP) segment sequences. The blue bars on the nodes represent the 95% highest posterior densities of the branch heights (this is the time for divergence in months). The European and Japanese clade is highlighted in red. The x-axis represents the date in years. [file peerj-03-934-s007.png]

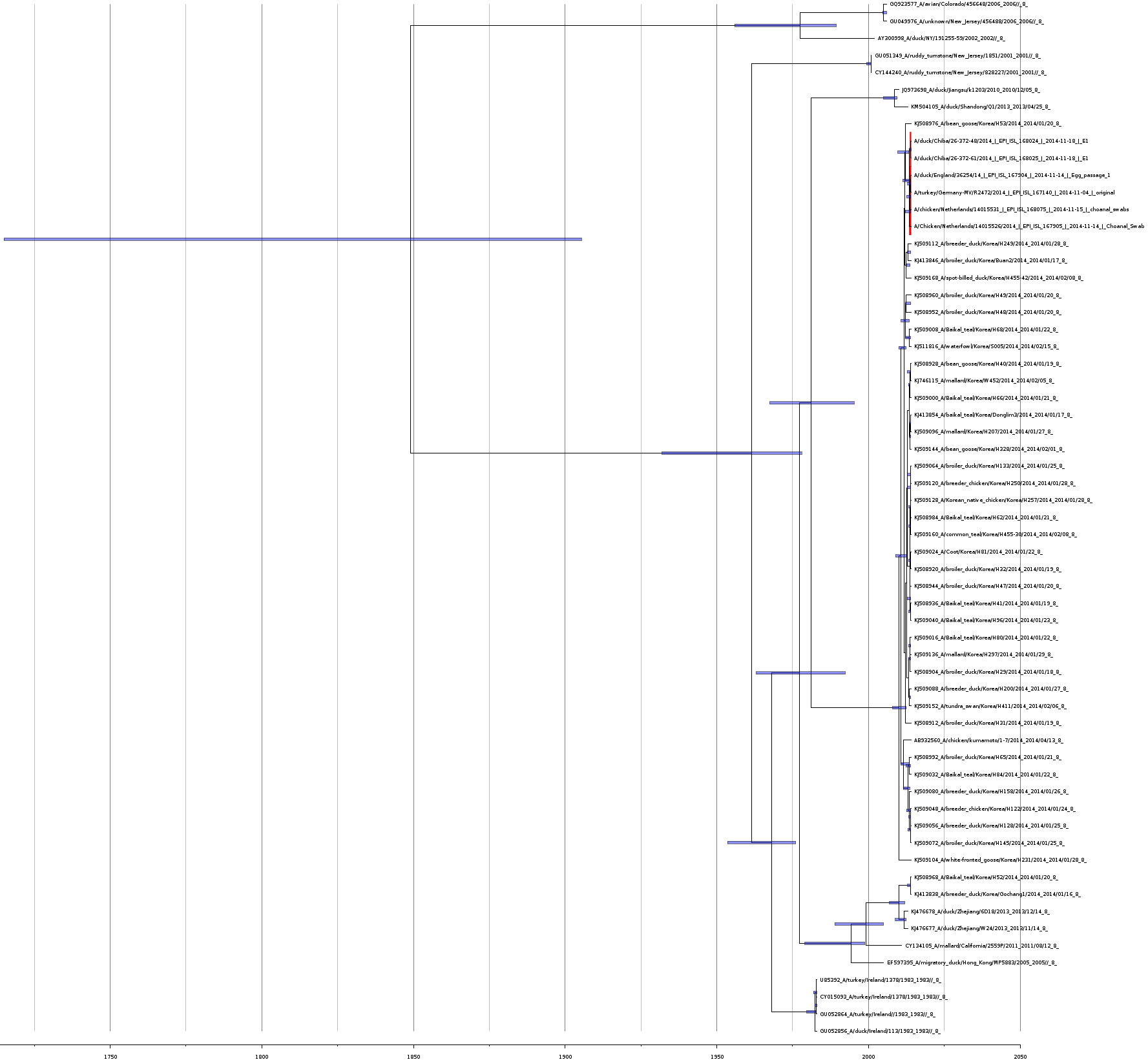

Supplement: Figure S8 — Bayesian coalescent gene tree for all of the H5N8 non-structural protein subunit (NS) segment sequences. The blue bars on the nodes represent the 95% highest posterior densities of the branch heights (this is the time for divergence in months). The European and Japanese clade is highlighted in red. The x-axis represents the date in years. [file peerj-03-934-s008.png]

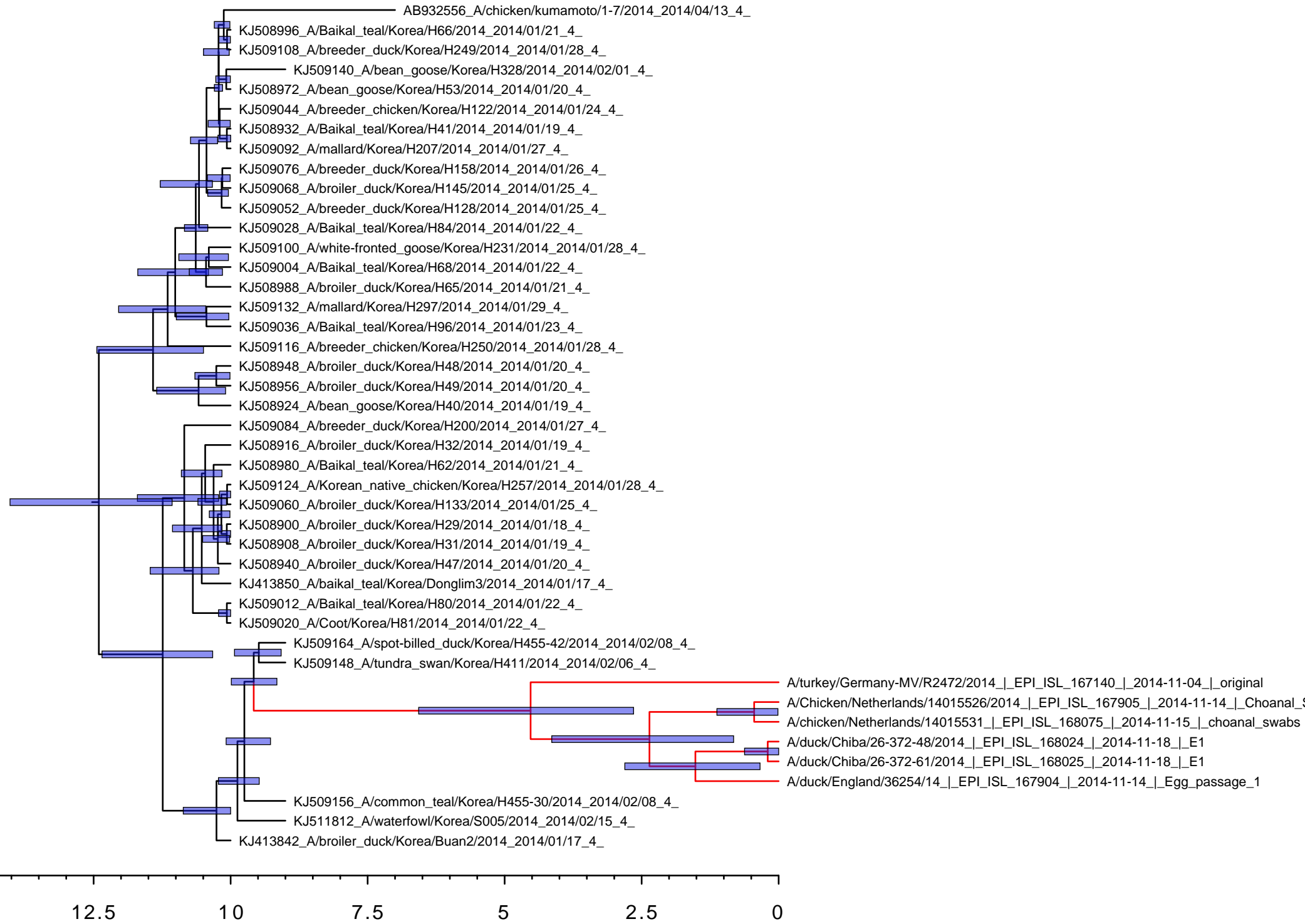

Supplement: Supplemental Information 9 [file peerj-03-934-s009.zip › H5N8_Analysis/HA_final_tree.pdf]

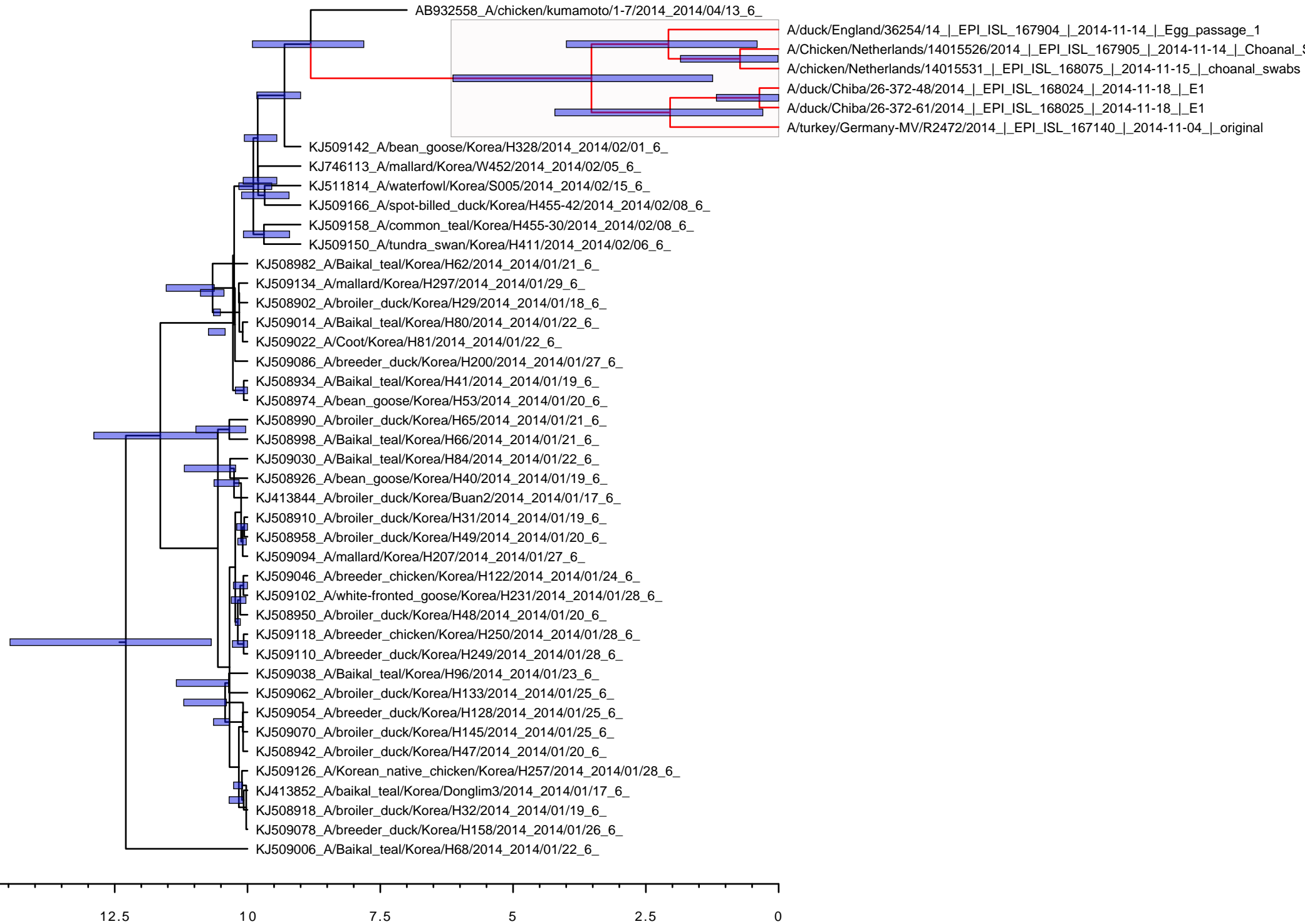

Supplement: Supplemental Information 9 [file peerj-03-934-s009.zip › H5N8_Analysis/NA_final.tree.pdf]
